# Hydrogen diffusion and uptake in nickel Alloy 625 under cathodic protection conditions

**DOI:** 10.1016/j.heliyon.2024.e33924

**Published:** 2024-07-02

**Authors:** Xu Lu, Roy Johnsen

**Affiliations:** Department of Mechanical and Industrial Engineering, Norwegian University of Science and Technology, Richard Birkelands vei 2B, N-7491, Trondheim, Norway

**Keywords:** Nickel Alloy 625, Hydrogen, Diffusion, Cathodic protection, Permeation

## Abstract

Hydrogen diffusion and uptake in nickel Alloy 625 under cathodic protection potential (−1050 mV_Ag/AgCl_) and temperature (10 °C) were studied using electrochemical permeation tests. It is the first time hydrogen permeation of nickel alloy at a temperature lower than room temperature was investigated. The results revealed that the effective diffusivity of hydrogen $D_{\text{eff}}$ at −1050 mV_Ag/AgCl_ varied from 1.81 to 2.86 × 10^−15^ m^2^/s across the temperature range of 10–23 °C. The effective subsurface hydrogen concentration $C_{sub}$ was influenced by both the applied temperature and overpotential. Particularly, the change in $C_{\text{sub}}$ at 10 °C is dependent on the hydrogen absorption efficiency affected by the surface coverage fraction of hydrogen. Furthermore, the hydrogen fugacity on the sample surface $f_{H_{2}}$, the applied overpotential, and the temperature have been successfully cross correlated to interpret hydrogen evolution and adsorption. It was demonstrated that $f_{H_{2}}$ primarily changed with the applied overpotential, while the temperature affected the gradient of $f_{H_{2}}$ during the potential increment. The current study provides valuable insights for industries, assisting in the prediction of hydrogen absorption and hydrogen-assisted failures in subsea nickel alloy components.

## Highlights

• Hydrogen diffusion and uptake are measured under cathodic protection conditions.
• $D_{eff}$ increases with temperature and the applied potential.
• Subsurface hydrogen concentration is dependent on the hydrogen absorption efficiency.
• Hydrogen fugacity $f_{H_{2}}$ increases with overpotential.
• Temperature affects the gradient of $f_{H_{2}}$ as the overpotential increases.

## Nomenclature

HSC: hydrogen assisted cracking
CP: cathodic protection
SCE: saturated calomel electrode
SEM: scanning electron microscopy
EDS: electron dispersive spectroscopy
SHE: standard hydrogen electrode
$D_{\text{eff}}$: effective diffusion coefficient
$C_{\text{sub}}$: subsurface hydrogen concentration
$f_{H_{2}}$: hydrogen fugacity
$t_{\text{lag}}$: lag time
$i_{\infty }$: steady-state current density
$M_{H}$: molar mass of hydrogen
ρ: density
R: gas constant
T: temperature
η: overpotential
$S_{H}$: solubility of hydrogen

## 1 Introduction

Nickel-based alloys have prominent mechanical properties with excellent corrosion resistance and have been widely used for downhole equipment, fasteners, and screws in the oil and gas industries for many years. These alloys, presumed to be immune to hydrogen stress cracking (HSC), have been reported to fail due to HSC after 20 years of subsea applications [[1], [2], [3]]. Typically, when nickel alloys are coupled to carbon steel (*i.e.*, pipes) that require cathodic protection (CP), hydrogen produced on the metal surface can be absorbed by the component. The occlusion of hydrogen led to material degradation, in particular, the loss of ductility and strength, leading to unexpected catastrophic failures. The underlying mechanisms behind this are rather complex due to multifaceted reasons. One of the widely discussed aspects is the effect of microstructure [[4], [5], [6], [7], [8], [9], [10]]. Besides the desired strengthening phases, *i.e.*, face-centered Ni_3_(Al, Ti) γ’ (L1_2_ ordered) and body-centered Ni_3_Nb γ’’ (D0_22_ ordered), other secondary phases, such as δ phase (Ni_3_Nb), η phase (Ni_3_Ti), σ phase/F phase, carbides and carbonitrides are frequently reported [[11], [12], [13], [14]]. The latter phases serve as effective trapping sites for hydrogen atoms and promote HSC under stress conditions. Another vital point is hydrogen uptake and diffusion, which determine the hydrogen concentration and the penetration depth of hydrogen in a specific environment. A well-accepted approach to probe hydrogen diffusion and uptake is the hydrogen permeation test (Devanathan-Stachursky method) based on ASTM G148 [15,16]. To date, the diffusivity, permeability, and trapping behavior of hydrogen in nickel alloys have been discussed in a wide range of nickel alloys, such as IN718 [[17], [18], [19]], IN625 [20], and Monel K-500 [21]. Diffusion equations were documented enabling the prediction of hydrogen diffusivity at different temperatures. However, studies on pure nickel [22,23], nickel alloys [20], and steels [[24], [25], [26]] have demonstrated that the microstructure, surface condition, and testing parameters (*i.e.*, current density, potential) would affect the permeation behavior of hydrogen. It is necessary to investigate hydrogen uptake and diffusion of the alloy in a case-to-case study. In addition, most studies were conducted at elevated temperatures to compensate for the low diffusion rate of hydrogen (∼10^−15^ m^2^/s). For Alloy 625 that are currently installed in the subsea pipeline system under low-temperature environment (∼10 °C), permeation testing on this alloy akin to the realistic condition regarding both the applied potential and temperature is essential. This information is scarcely documented in the literature, while it is important when evaluating the HSC problem of subsea components. Crucial questions that need in-depth investigation encompass: (a) is the hydrogen permeation behavior at low-temperature (∼10 °C) environment can be extrapolated from the results at higher temperatures? (b) how hydrogen fugacity alters with overpotential? and (c) does temperature affect hydrogen fugacity? To complement the pioneering work and to answer the above research questions, particular attention was drawn to the effect of temperature and cathodic potential on hydrogen permeation close to real subsea conditions in an Alloy 625. Additionally, the surface hydrogen fugacity was evaluated, providing extra information on hydrogen activity on the alloy surface during cathodic polarization.

## 2 Experimental

The chemical composition of the studied Alloy 625 can be found in Ref. [20]. The alloy was forged, rolled, and annealed at 885 °C for 1 h followed by water quenching. Disc samples were machined out of the alloy bar with a diameter of 16 mm. Afterward, the samples were ground with SiC paper till #4000 and further prepared with 3 and 1 μm diamond pastes. To achieve a deformation-free surface for microstructure characterization, silica suspension was used as the final polishing step. The final thickness of the sample was 60 μm to reduce the permeation time. To study the hydrogen permeation behavior, a double-wall Devanathan-Stachursky electrolytic permeability cell was used. On the hydrogen detection side, a constant anodic potential at −99 mV vs. mercury-mercurous sulfate reference electrode (MSE, equals +300 mV vs. saturated calomel electrode (SCE)) in the 0.1 M NaOH solution (pH 12.78) was applied. On the hydrogen charging side, 0.1 M NaOH solution (pH 12.80) with an addition of 0.2 g/L thiourea was used. A stepwise polarization potential was applied starting at −1050 mV_Ag/AgCl_ and decreased at −100 mV stepwise till −1350 mV_Ag/AgCl_. The temperatures for the permeation tests were controlled at 10 and 23 °C using a circulating water bath with a cooler. It is worth mentioning that even though the applied electrolyte is not identical to seawater, the intention is to study hydrogen permeation in an environment akin to real subsea application regarding the temperature and cathodic protection potential. In addition, the microstructure of the alloy was characterized using a high-resolution Quanta FEG 650 scanning electron microscope (SEM, Thermo Fisher Scientific Inc., USA). Electron dispersive spectroscopy (EDS) was employed to characterize the grain boundary precipitate.

## 3 Results and discussions

**Fig. 1:**
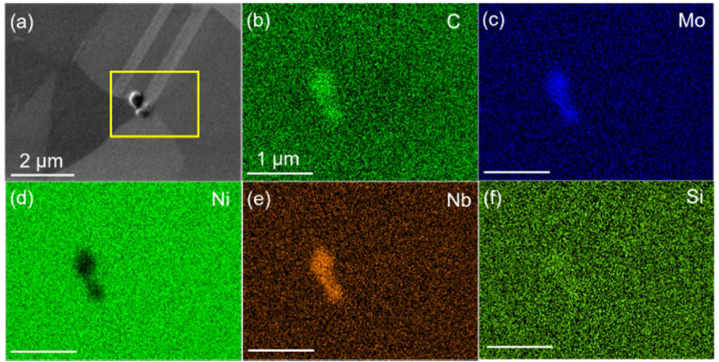
(a) Micrograph of the studied nickel alloy showing grain boundary carbides; (b)–(f) the corresponding EDS mapping on the area highlighted in (a) showing enrichment in C, Mo, Nb, Si, and depletion in Ni compared with the matrix.

**Fig. 2:**
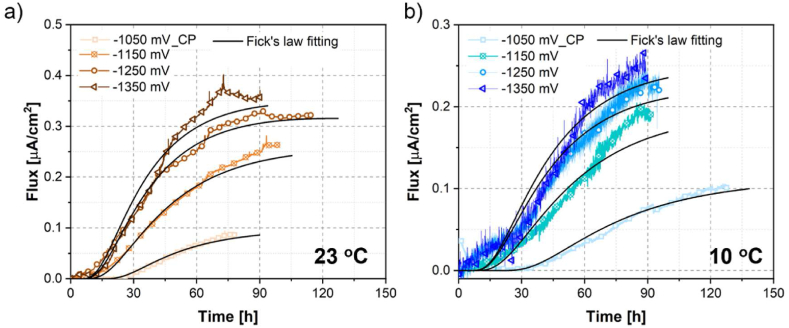
Electrochemical permeation profiles at (a) 23 °C and (b) 10 °C under different cathodic potentials: 1050, −1150, −1250, −1350 mV_Ag/AgCl_, respectively. The theoretical curves from Fick's law fitting were included.

The SEM micrograph of the studied alloy (Fig. 1(a)) shows a polycrystalline face-centered cubic structure with annealing twins in the grains. The average grain size is 7.96 μm with a twin fraction of 52.4 %. The grain boundary precipitate is highlighted and the corresponding EDS mapping manifests enrichment in C, Mo, Nb, Si, and depletion in Ni compared with the matrix (Fig. 1(b)–(f)). The precipitate has been reported to be M_6_C carbides in the previous study by the authors [20]. Hydrogen permeation profiles at different temperatures and various cathodic charging potentials, *i.e.*, −1050, −1150, −1250, −1350 mV_Ag/AgCl_, are plotted in Fig. 2. At a fixed temperature, a decrease in the charging potential leads to both a time reduction for hydrogen detection and an elevation in the permeation flux. When comparing different temperatures, the maximum hydrogen flux is typically reduced by lowering the temperature. More noise was recorded at a lowered temperature due to the high noise-to-signal ratio. The fluctuations might be due to the variation of the testing environment in the long-term test, such as the pH of the electrolyte, the concentration of the poison, and the stability of the reference electrode. Detailed explanations are elaborated in the following sections. Theoretical curves from Fick's law fitting [27] are shown in Fig. 2. An acceleration in diffusion was observed for the experimental curves when the permeation approached the steady state. It should be mentioned here that the potential of −1050 mV_Ag/AgCl_ at 10 °C represents the environmental conditions close to CP in northern Europe subsea applications. The corresponding applied cathodic current density (mA/cm^2^) is summarized in Table 1. At −1050 mV_Ag/AgCl_, the current densities (0.0242−0.0380 mA/cm^2^) are close to the values reported in the literature for a typical CP in seawater [28].

**Table 1:** Cathodic current densities and $D_{\text{eff}}$ values at different testing temperatures and applied potentials. $D_{\text{eff}}$ values from Fick's law fitting were included.

Applied potential (mV_Ag/AgCl_)	−1050	−1150	−1250	−1350
Temperature (°C)	Cathodic current density (mA/cm^2^)			
10	0.0380	0.180	0.454	1.151
23	0.0242	0.113	0.344	1.077

Temperature (°C)	$D_{\text{eff}}$ ( × 10^−15^ m^2^/s)			
	Experimental/Fick's law			
10	1.81/2.10	2.26/2.46	2.65/3.09	3.13/3.25
23	2.86/3.16	3.08/3.18	4.02/3.99	3.81/4.21

To evaluate the effective diffusion coefficient $D_{\text{eff}}$ for hydrogen, the time-lag method ($t_{\text{lag}}$, Eq. (1)) was adopted [29]:

(1) $$D_{\text{eff}\left(t_{\text{lag}}\right)}=\frac{L^{2}}{6t_{\text{lag}}}$$

where $t_{\text{lag}}$ is the time (s) for the hydrogen flux to reach 0.63 times the steady-state current density $i_{\infty }$ (μA/cm^2^). In addition, at an equilibrium condition, the effective subsurface hydrogen concentration $C_{\text{sub}}$ (wppm) at the charging side is proportional to $i_{\infty }$ at the detection side [24]:

(2) $$C_{\text{sub}}=\frac{i_{\infty }L}{FD_{\text{eff}}}\;\frac{M_{H}}{\rho }\;10^{6}$$

where F is the Faraday constant (96485.3 A⋅s/mol), $M_{H}$ is the molar mass of hydrogen (1 g/mol), and ρ is the density of the studied alloy (8.37 g/m^3^).

The $D_{\text{eff}}$ values at different temperatures and cathodic potentials are summarized in Table 1. It needs to be mentioned that due to the strong noises in the permeation curves at 10 °C, the relative steady-state flux was determined in a way that the current fluctuation is within 15 % of the recorded average signal for more than 5 h. Based on the values in Table 1, a pronounced escalation of $D_{\text{eff}}$ with temperature was observed, which could be easily verified by the diffusion equation [20]. While $D_{\text{eff}}$ increases only slightly by lowering the applied potential. Normally the diffusion coefficient of hydrogen is an intrinsic property of the metal, which should be only dependent on the temperature. However, variation in $D_{\text{eff}}$ by changing the charging conditions has also been reported in nickel alloys and steels depending on the surface hydrogen evolution reactions and the hydrogen trapping behavior in materials [20,24]. On one aspect, the increase of $D_{\text{eff}}$ with higher overpotential at all test temperatures can be attributed to the enhanced hydrogen evolution on the metal surface. On the other aspect, more trapping sites were filled at lower potentials, rendering the lattice diffusion to play a dominant role in hydrogen diffusion. The $D_{\text{eff}}$ values from theoretical Fick's law fitting are summarized in Table 1. The experimental results are deviated from that calculated from Fick's law. This can be caused by the lattice expansion induced by hydrogen incorporation, leading to the modification of the stress gradient. Such stress gradient accelerated the hydrogen diffusion [30]. It needs to mention that the accelerated hydrogen diffusion at a later stage of the permeation could be due to the hydrogen-assisted vacancy formation, as proposed by Feaugas et al. [30], who reported similar results in nickel single crystals. By taking into account the stress field associated with vacancies, or clusters of vacancies, a modified Fick's model could better fit the experimental curves [30]. The acceleration effect in diffusion occurs especially at the later stage of permeation, which indicates that the hydrogen-assisted vacancy formation could be promoted substantially after the hydrogen concentration is higher than a critical value. Nevertheless, the slight deviation in $D_{\text{eff}}$ between the two methods does not influence the conclusions drawn on the effect of the temperature and applied potential. In addition, it has been well-discussed that the scatter from duplicate tests is indeed important for the permeation tests [27]. The inherent scatter could render $D_{\text{eff}}$ a range of values. However, our study shows a consistency of $D_{\text{eff}}$ with literature on pure Ni [30] and nickel alloys [20] regarding the effect of the applied potential and temperature. It is reasonable to deduce that the main conclusions would not be influenced by the possible scatter from duplicated tests provided that the tests are conducted consistently.

**Fig. 3:**
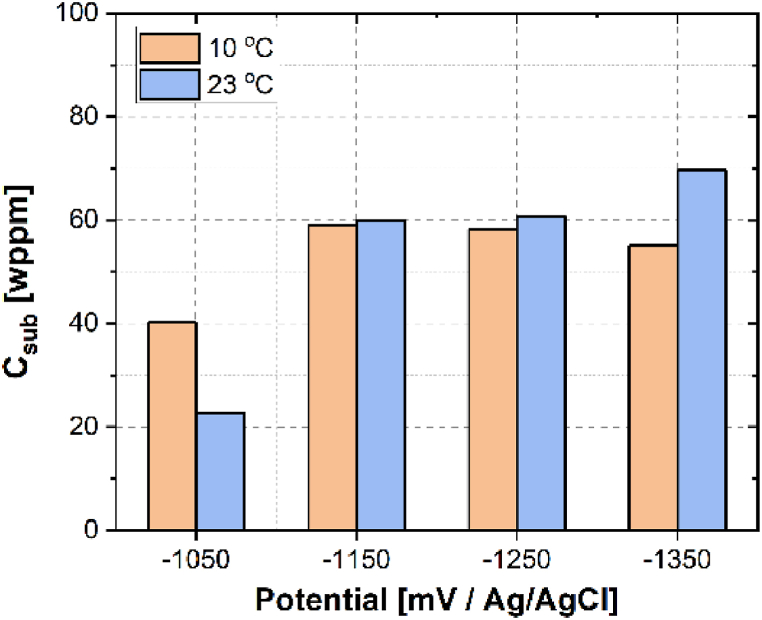
The effect of cathodic charging potential and testing temperatures on the subsurface hydrogen concentration $C_{\text{sub}}$.

In addition, the effect of temperature and the applied potential on $C_{\text{sub}}$ is displayed in Fig. 3, It was noticed that the change of $C_{\text{sub}}$ is attributed to the synergistic effect of temperature, potential, and $D_{\text{eff}}$. At −1050 mV_Ag/AgCl_, where hydrogen evolution reaction is slow on the sample surface, $C_{\text{sub}}$ presents no trend with temperature even though the $D_{\text{eff}}$ values are strongly influenced. In this condition, traps were gradually filled. When the applied potential decreased from −1150 to −1350 mV_Ag/AgCl_, a higher temperature ended up with larger $C_{\text{sub}}$, while lowering the potential led to different behavior in hydrogen adsorption. In this potential range, hydrogen evolution reaction is highly promoted, as can be proven by the cathodic current densities in Table 1. Hydrogen atoms produced on the sample surface can either be absorbed in the alloy or recombine as hydrogen molecules and leave the surface. At a higher temperature of 23 °C, $C_{\text{sub}}$ increased due to the higher $D_{\text{eff}}$ and the enhanced hydrogen production at the surface. Superabundant hydrogen atoms would leave the sample as molecules. At 10 °C, $C_{\text{sub}}$ decreased slightly with lowered potential even though $D_{\text{eff}}$ increased. It could be due to the reduced fraction of surface coverage by hydrogen at a lower temperature. This phenomenon is highly dependent on the hydrogen mobility in the electrolyte and the surface coverage fraction by hydrogen atoms. One possible reason is that hydrogen bubbles generated on the sample surface blocked the effective area for hydrogen absorption. However, those bubbles are difficult to be stripped away due to the low thermodynamic mobility of hydrogen molecules at low temperatures [31]. Therefore, the amount of hydrogen entering the sample is decreased because of the reduced area to produce hydrogen. It has been reported that when the permeation temperature is 60 °C, a reduction in $C_{\text{sub}}$ was also observed at cathodic current densities higher than 40 mA/cm^2^ [20]. However, the mechanism is different. At 60 °C, the lowered hydrogen solubility in the sample was attributed to the prevailing Tafel or Heyrovsky reactions compared with the Volmer reaction, where hydrogen atoms combining into molecules and leaving the sample was highly promoted. It is thus recommended that, at low-temperature subsea applications, special attention should be paid to the effect of cathodic potential on hydrogen absorption. It needs to be addressed that the surface conditions before and after the permeation test were carefully checked, and no oxide layer was observed. In addition, consistency in both the sample preparation and testing was guaranteed so that the surface effect on the permeation behavior of hydrogen could be excluded.

Hydrogen fugacity $f_{H_{2}}$ is an important factor reflecting the hydrogen activity on the sample surface. It refers to the effective pressure of a real gas. The applied overpotential on the cathodic side η can be linked to $f_{H_{2}}$ based on the classic Nernst equation [32]:

(3) $$f_{H_{2}}=Aexp\left(\frac{-\eta F}{BRT}\right)$$

**Fig. 4:**
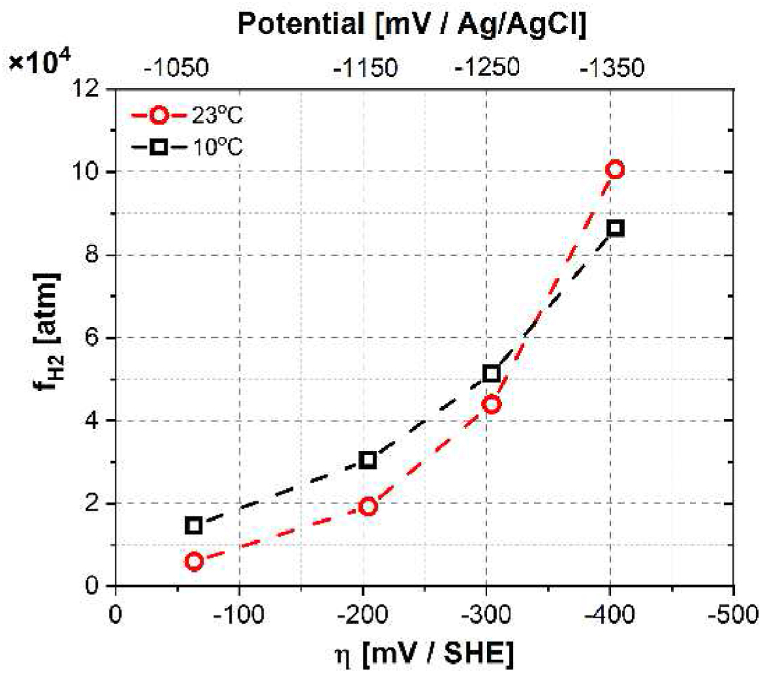
Hydrogen fugacity $f_{H_{2}}$ as a function of overpotential η at permeation temperatures of 10 and 23 °C.

Where A and B are constants, R is the gas constant as 8.31446 J/(K × mol), T is the temperature in K and η is the overpotential (mV vs. standard hydrogen electrode (SHE)) in reference to the equilibrium potential during hydrogen evolution reaction at 1 atm [25]. By correlating $f_{H_{2}}$ with $C_{\text{sub}}$ based on Sievert's law, $i_{\infty }$ can be reformulated as a function of $f_{H_{2}}$:

(4) $$i_{\infty }=\frac{FD_{\text{eff}}\;S_{H}}{L}\frac{\rho }{M_{H}\times 10^{6}}\sqrt{\left(Aexp\left(\frac{-\eta F}{BRT}\right)\right)}$$

where $S_{H}$ (wppm H atm^−1/2^) is the solubility of hydrogen in nickel alloys [33]. $M_{H}$ is the molar mass of hydrogen (1 g/mol), and ρ is the density of the studied alloy (8.37 g/m^3^). The parameters A and B can be deduced based on the fitting between $\ln\;i_{\infty }$ and η, and the results are summarized in Table 2. Details of the derivation can be found in Ref. [20]. Fig. 4 shows the evolution of $f_{H_{2}}$ as a function of the temperature and η. As expected, $f_{H_{2}}$ increases with increased overpotential. At CP potential (−1050 mV_Ag/AgCl_), $f_{H_{2}}$ reaches about 0.59–1.46 × 10^4^ atm, and it increases to 6−17 times higher at −1350 mV_Ag/AgCl_. At a constant overpotential, no clear trend was observed between $f_{H_{2}}$ and the temperature. However, higher temperature manifests a steeper $f_{H_{2}}$ augmentation gradient by increasing the overpotential. This indicates that hydrogen evolution reactions could be promoted more significantly at higher temperatures. Besides the effect of temperature and potential, the type of electrolytic solution has also been demonstrated to affect $f_{H_{2}}$ [25]. A spread of $f_{H_{2}}$ was reported between alkaline 0.1 M NaOH and acidic Na_2_SO_4_ solutions, which could be attributed to different true surface areas at a particular cathodic charging condition. Surface oxide, which can easily form on steel surfaces, was claimed to reduce $f_{H_{2}}$ by lowering the hydrogen surface coverage [34]. However the surface oxide can be partially removed by cathodic pre-charging [25]. In addition, calcareous deposition is also important for hydrogen permeation as it might act as a barrier for hydrogen entry like surface oxide [35]. Therefore, for subsea applications, it is necessary to consider the synergy effect of several factors, such as temperature, surface oxide, calcareous deposition, seawater composition, *etc.*, when assessing the hydrogen permeability of an alloy.

**Table 2:** The calculated A and B values at different temperatures.

Temperature (°C)	A	B
10	10515.95	7.87
23	3535.59	4.75

Indeed, the permeability of hydrogen in the IN625 has been investigated by employing the gaseous permeation tests at high temperatures up to about 1200 K [[36], [37], [38]]. Reformulating the data from Mitchnel et al. [36] enables the diffusivity of hydrogen to be expressed as $D=8.32\times 10^{-7}\;\exp\;\left(\frac{-49.21\;\text{kJ}/\text{mol}}{\text{RT}}\right)$ m^2^/s. The extrapolations of the equation yield the hydrogen diffusivities of 1.62 × 10^−15^ m^2^/s and 6.97 × 10^−16^ m^2^/s for the temperatures of 23 and 10 °C, respectively. The extrapolated diffusivities from high-temperature tests are biased to the lower values, and this trend is exemplified by lowering the temperature. Differences in values could be caused by variations in the microstructure (grain size, precipitates), testing media (gaseous vs electrochemical), sample thickness, and surface conditions during hydrogen uptake. This indicates that the temperature effect on the hydrogen uptake and diffusion might be overestimated when the temperature is below room temperature, with the noises considered. Therefore, additional evaluation is essential when handling the data extrapolated from high-temperature tests for low-temperature predictions. The novelty of the work resides in the following aspects: (a) it is the first time hydrogen permeation behavior of nickel alloy was studied under the temperature lower than room temperature, *i.e.*, at 10 °C, (b) the hydrogen fugacity on the sample surface, the applied overpotential, and the temperature have been successfully cross correlated to interpret hydrogen evolution at low temperature, (c) the current study provides valuable insights for industries, assisting in the prediction of hydrogen absorption and hydrogen-assisted failures in subsea nickel alloy components.

## 4 Conclusions

In this study, hydrogen diffusion and uptake behavior under cathodic protection conditions in a nickel Alloy 625 was investigated using electrochemical permeation tests. The results revealed that the effective diffusivity of hydrogen $D_{\text{eff}}$ under typical cathodic protection potential (−1050 mV_Ag/AgCl_) changed from 1.81 to 2.86 × 10^−15^ m^2^/s depending on the applied temperatures. The effective subsurface hydrogen concentration $C_{\text{sub}}$ was influenced by both the temperature and applied overpotential. Especially, at a lower temperature (10 °C), the change in $C_{\text{sub}}$ is dependent on the hydrogen absorption efficiency affected by the surface coverage fraction of hydrogen and hydrogen molecule mobility. In addition, the results demonstrate that the hydrogen fugacity $f_{H_{2}}$ changed primarily with the overpotential, while the temperature affects the gradient of $f_{H_{2}}$.

## Data availability statement

The raw/processed data required to reproduce the above findings cannot be shared at this time as the data also forms part of an ongoing study.

## CRediT authorship contribution statement

**Xu Lu:** Writing – original draft, Validation, Methodology, Investigation, Formal analysis, Data curation, Conceptualization. **Roy Johnsen:** Writing – review & editing, Supervision, Resources, Funding acquisition.

## Declaration of competing interest

The authors declare that they have no known competing financial interests or personal relationships that could have appeared to influence the work reported in this paper.
